# Resistance to β-Lactam Antibiotics Conferred by Point Mutations in Penicillin-Binding Proteins PBP3, PBP4 and PBP6 in *Salmonella enterica*

**DOI:** 10.1371/journal.pone.0097202

**Published:** 2014-05-08

**Authors:** Song Sun, Maria Selmer, Dan I. Andersson

**Affiliations:** 1 Department of Medical Biochemistry and Microbiology, Uppsala University, Uppsala, Sweden; 2 Department of Cell and Molecular Biology, Uppsala University, Uppsala, Sweden; Institut National de la Recherche Agronomique, France

## Abstract

Penicillin-binding proteins (PBPs) are enzymes responsible for the polymerization of the glycan strand and the cross-linking between glycan chains as well as the target proteins for β-lactam antibiotics. Mutational alterations in PBPs can confer resistance either by reducing binding of the antibiotic to the active site or by evolving a β-lactamase activity that degrades the antibiotic. As no systematic studies have been performed to examine the potential of all PBPs present in one bacterial species to evolve increased resistance against β-lactam antibiotics, we explored the ability of fifteen different defined or putative PBPs in *Salmonella enterica* to acquire increased resistance against penicillin G. We could after mutagenesis and selection in presence of penicillin G isolate mutants with amino-acid substitutions in the PBPs, FtsI, DacB and DacC (corresponding to PBP3, PBP4 and PBP6) with increased resistance against β-lactam antibiotics. Our results suggest that: (i) most evolved PBPs became ‘generalists” with increased resistance against several different classes of β-lactam antibiotics, (ii) synergistic interactions between mutations conferring antibiotic resistance are common and (iii) the mechanism of resistance of these mutants could be to make the active site more accessible for water allowing hydrolysis or less binding to β-lactam antibiotics.

## Introduction

The bacterial peptidoglycan, a three-dimensional, net-like mesh, is the major constituent of the cell wall [1]. Penicillin-binding proteins (PBPs) are a family of enzymes that are responsible for the polymerization of the glycan strand and the cross-linking between glycan chains [2]. These proteins are referred as PBPs due to their ability to bind to β-lactam antibiotics [3], [4] and based on their molecular weights, PBPs can be classified into two groups: low molecular weight PBPs and high molecular weight PBPs, each of which is subdivided into three classes based on amino-acid sequence similarities [5]. The penicillin-binding domains of PBPs function as DD-transpeptidases, which catalyze the final step of cell wall biosynthesis by cross-linking two strands of peptidoglycan, or DD-peptidases, which remove the C-terminal D-alanine from the peptidoglycan [2], [6]. Usually, these enzymes are acyl-serine transferases that catalyze their reactions by employing an active-site serine moiety as a nucleophile to attack the acyl-D-Ala-D-Ala portion of the peptidoglycan [3], [6]. These reactions are mediated by three specific functional motifs: SXXK, (S/Y)XN and (K/H)(S/T)G [5], [6]. The resistance-causing β-lactamases of class A, C and D contain the same three specific functional motifs and also employ an active-site serine residue to turn over β-lactam antibiotics [5], [7]. In terms of the reaction with β-lactam antibiotics, for both PBPs and β-lactamases, in the first acylation step, the active-site serine attacks the β-lactam ring present in these antibiotics forming a covalent acyl-enzyme complex. The second deacylation step is very fast with β-lactamases but extremely slow with PBPs [5].

Sequence, structural and catalytic analyses suggest that serine β-lactamases evolved from the more ancient PBPs [8]–[11] and several studies have shown that some PBPs display β-lactamase activity [12]–[15]. Previously, β-lactamase activity of a PBP subjected to point mutations or protein engineering based on the structural comparison of these two groups of enzymes [15]–[17] has been demonstrated. However, studies to date have not systematically examined the potential of all PBPs present in one organism to evolve resistance towards β-lactams by increasing β-lactamase activity.

Another, better defined, mechanism by which alterations in a PBP could confer resistance is by lowering the affinity of the drug to the active site, allowing the DD-transpeptidase activity to proceed without inhibition in presence of the drug. This mechanism has been described for several PBPs and is a common resistance towards β-lactam antibiotics in, for example, *Streptococcus penumoniae* [7], [18].

In this work, we explored the ability of fifteen different defined or putative PBPs in *Salmonella enterica var* Typhimurium LT2 to evolve increased resistance against penicillin-G. *Salmonella enterica var* Typhimurium LT2 is one *Salmonella* serovar that belongs to the *S. enterica* subsp. *enterica* subspecies and it will be referred as *Salmonella typhimurium* throughout the text. After screening of the fitness effect of PBP overexpression, seven PBPs, overexpression of which did not confer any significant fitness cost, were subject to two different directed evolution strategies. After constructing and cloning the mutagenesis libraries into the *Salmonella typhimurium* LT2 strains, for three PBPs, FtsI, DacB and DacC, corresponding to PBP3, PBP4 and PBP6, respectively, we isolated mutants with increased resistance against penicillin G. Our result suggest that PBPs can after only a few amino-acid substitutions confer increased resistance against many types of β-lactam antibiotics.

## Materials and Methods

### Construction of the Salmonella typhimurium expression plasmid pUCBAD-kan-PBP

The expression vector used in this study was constructed from pBAD30 [19]. pBAD30 is a tightly controlled expression vector regulated by the arabinose operon, for which the expression is induced by adding arabinose into the growth medium. Two-step modification of the original pBAD30 resulted in the plasmid pUCBAD-Kan where: i) the replication origin of pBAD30 was replaced with the pUC19 replication origin, which made pUCBAD-kan a high copy number plasmid, ii) the TEM-1 β-lactamase gene encoded in pUCBAD30 was replaced with the aminoglycoside 3′phosphotransferase (kanamycin-resistance) gene from pKD4.

Based on the protein gene predictions and functional annotation of the *Salmonella typhimurium* LT2 genome [20], fifteen gene products were annotated as putative penicillin-binding proteins in *Salmonella typhimurium* LT2: *ampH, dacA, dacB, dacC, dacD, mrcA, mrcB, mrdA, pbpC, pbpG, STM1836, STM1910, STM2478, ftsI, and yfeL*. Gene predictions were performed using two microbial gene prediction tools, GeneMark (http://opal.biology. gatech.edu /GeneMark/) and Glimmer (http://www.cbcb.umd.edu/software/glimmer/), and functional annotation was performed by searching the predicted protein sequence against the protein family database, pfam (http://pfam.sanger.ac.uk/), the COG database [21], and the protein localization prediction software, PSORT [22] described in [20]. Each of these candidate PBP encoding genes was PCR-amplified from *Salmonella typhimurium* genomic DNA using a forward primer carrying an *Eco*RI or *Sma*I (for the *ftsI* gene) restriction enzyme site and a reverse primer containing an *Xba*I site. The PCR fragments were digested and cloned into pUCBAD-kan. The resulting plasmid, designated as pUCBAD-kan-PBP was then isolated and subsequently transformed into the *Salmonella typhimurium* LT2 strain. All primers used are listed in Table S1 in File S1.

### Hydroxylamine mutagenesis

Two micrograms plasmid DNA was mixed with 100 µl 0.1 M phosphate buffer containing 1 mM EDTA (pH  = 6.0) and 80 µl hydroxylamine solution (0.35 g hydroxylamine hydrochloride, 0.56 ml 4 M NaOH, 4.5 ml H_2_O, pH  = 6.0, 1 mM EDTA) and incubated at 70°C for 120 minutes. Plasmid DNA was then purified from hydroxylamine using Fermentas GeneJET Gel Extraction Kit and subsequently electroporated into the *Salmonella typhimurium* LT2 strain.

### Error prone PCR (ep-PCR) mutagenesis

Error prone PCR was performed on 30 ng template plasmid for 25 cycles using GeneMorph II Random Mutagenesis Kit from Agilent Technologies. To clone the libraries, 400 ng of digested PCR product was ligated to 400 ng of digested pUCBAD-Kan at 16°C overnight using T4 DNA ligase. The ligation mixture was purified with SureClean Kit (Bioline) and dissolved in 10 µl of water. The ligated DNA was transformed into MegaX DH10B T1R Electrocomp Cells (Life Technologies) by electroporation. All the MegaX DH10B T1R transformants were subsequently propagated for 24 hrs at 37°C in 20 ml LB medium with kanamycin. Plasmid DNA was prepared from the overnight culture and used to transform the *Salmonella typhimurium* LT2 strain by electroporation.

### Selection and characterization of mutants

For both hydroxylamine and ep-PCR mutagenesis libraries constructed in the *Salmonella typhimurium* LT2 strain, ∼2×10^7^ cells were plated on each of ten LB agar plates (∼2×10^6^ cells on each plate) with kanamycin (50 mg/L), arabinose (2 mM) and increasing concentrations of penicillin-G (8, 12 and 16 mg/L), and incubated at 37°C for 48 hours. The resistant colonies were subsequently streaked onto the original selection plates and the plates lacking arabinose, and those clones surviving only on the original plates with arabinose were selected for further characterization as the arabinose induction dependent growth indicated that the resistance to β-lactam antibiotics was conferred by expression of the PBP encoding genes.

### Determination of minimum inhibitory concentrations

The minimum inhibitory concentrations (MICs) of penicillin-G for all tested strains in this work were measured by macrobroth dilution test. A serial dilution of antibiotics were made in LB broth with kanamycin (50 mg/L) and arabinose (2 mM) dispensed in test tubes: penicillin-G (2, 4, 6, 8, 10, 12, 14, 16, 18, 20, 22, 24, 26, 28, 30 mg/L); ampicillin (0.5, 1, 1.5, 2, 2.5, 3, 3.5, 4, 4.5, 5 mg/L); cephalothin (1, 1.5, 2, 2.5, 3, 3.5, 4, 4.5, 5, 6, 7, 8, 9, 10 mg/L); cefuroxime (2, 3, 4, 5, 6, 7, 9, 11, 13, 15, 18, 21, 23 mg/L); cefotaxime (0.025, 0.05, 0.075, 0.1, 0.125, 0.15, 0.175, 0.2 mg/L). The final volume in test tubes is 1 ml and the final inoculum size is 5×10^5^ colony-forming units (cfu) ml^−1^. MIC was determined as the lowest concentration of antibiotic that inhibited growth of bacteria as assessed visually (no turbidity). Each MIC value reported is an average of two independent experiments performed in duplicate. Throughout the paper, resistance is defined as a significant reduction in drug susceptibility in the mutant as compared to the ancestral starting strain (i.e. resistance is not defined with regard to clinical breakpoints and the S, I, R system).

### Site-directed mutagenesis

Site-directed mutagenesis was performed by using the Thermo Scientific Phusion Site-Directed Mutagenesis Kit according to manufacturer's instructions. The pUCBAD-kan::PBP(wt) plasmid was amplified using phosphorylated primers that introduce the desired changes followed by a quick ligation reaction. The resulting plasmid was then transformed into NEB 5-alpha competent *E. coli* cells (New England Biolabs). All primers used are listed in Table S1 in File S1.

## Results and Discussion

### Effect of PBP-encoding gene expression on fitness and penicillin-G susceptibility in *Salmonella typhimurium*

Fifteen genes encoding defined or putative PBPs in *Salmonella typhimurium* LT2 genome (*ampH, dacA, dacB, dacC, dacD, mrcA, mrcB, mrdA, pbpC, pbpG, STM1836, STM1910, STM2478, ftsI, and yfeL*) were cloned in the pUCBAD-kan vector and transformed into the *Salmonella typhimurium* LT2 strain as described in Materials and Methods. Since using bacterial strains with severe growth defect could lead to selection of mutants with improved growth instead of increased resistance against antibiotics, the fitness impact of PBP-overexpression was examined for the fifteen strains expressing a PBP gene from pUCBAD-kan plasmid by streaking them on LB agar plates containing 2 mM arabinose and 50 mg/L kanamycin followed by 24-hour incubation at 37°C. The fitness of a bacterial strain was assessed by colony size. Compared to the *Salmonella typhimurium* LT2 strain carrying the empty pUCBAD-kan plasmid, seven strains expressing the PBP genes (*ampH*, *dacA*, *dacB*, *dacC*, *pbpC*, *pbpG* and *ftsI*) from the plasmid did not exhibit any significant growth defect and were selected for further investigation. These seven strains and the control strain with the empty plasmid were grown overnight at 37°C in the presence of 2 mM arabinose and MICs of penicillin G were determined (Table 1). No significant increase in MIC of penicillin G was observed for the seven strains under arabinose induction as compared to the control strain.

**Table 1:** Genotypes and MICs of the strains carrying the wild type PBPs used.

Strain No.	1Genotype	MIC of penicillin-G (mg/L)	
		No induction	Induction (2mM arabinose)
DA16007	Sty LT2/pucBAD-kan-*ampH*	5	5
DA16008	Sty LT2/pucBAD-kan-*dacA*	5	6
DA16009	Sty LT2/pucBAD-kan-*dacB*	5	6
DA16010	Sty LT2/pucBAD-kan-*dacC*	5	6
DA16015	Sty LT2/pucBAD-kan-*pbpC*	5	5
DA16016	Sty LT2/pucBAD-kan-*pbpG*	5	5
DA16020	Sty LT2/pucBAD-kan-*ftsI*	5	5
DA16006	Sty LT2/pucBAD-kan	4	4

1 Sty  =  *Salmonella typhimurium.*

### Isolation and characterization of penicillin-G resistant *Salmonella typhimurium* transformants

A library of mutated PBP-carrying plasmid DNA was generated for each of the seven selected strains (each carrying one of the PBP-encoding genes *ampH*, *dacA*, *dacB*, *dacC*, *pbpC*, *pbpG* and *ftsI* in plasmid pUCBAD-kan) by hydroxylamine mutagenesis as described in Materials and Methods. These seven libraries of randomly mutagenized plasmids were then transformed into the *Salmonella typhimurium* LT2 strain and each of the resulting libraries (designated as the library ampH_HM, dacA_HM, dacB_HM, dacC_HM, pbpC_HM, pbpG_HM and ftsI_HM) contained ∼10^4^ transformants. Variants with increased resistance against penicillin-G were selected from each library on LA plates supplemented with penicillin-G (8 and 12 mg/L) and 2mM arabinose. To determine whether the expression of the PBP variant was responsible for the increased resistance, resistant colonies were restreaked on LA plates containing the same concentrations of penicillin-G as used for selection with or without 2 mM arabinose. Fourteen clones, among which nine, one and four were selected from the libraries ftsI_HM, dacB_HM, and dacC_HM respectively, exhibited arabinose-dependent resistance against penicillin-G. Only from clones where growth was dependent on presence of arabinose, the plasmids were transformed to new cells followed by determination of the MICs against penicillin-G. The nine tranformants carrying the *ftsI* gene showed ∼2–3 fold increased resistance against penicillin G whereas the resistance level of the other five transformants carrying the *dacB* or *dacC* gene was indistinguishable from the strain expressing the corresponding wild PBP gene (Table 2). The ribosome binding site and coding region of the corresponding PBP genes were sequenced for these fourteen resistant transformants. Three different single amino-acid substitutions in the FtsI protein were identified for the nine resistant tranformants isolated from the ftsI_HM library and no mutations were found in the *dacB* and *dacC* genes for the other five transformants (Table 2).

**Table 2:** MICs of Penicillin-G and identified mutations for transformants with increased resistance.

Strain No.	Mutant PBPs	Mutagenesis strategy	1MIC of Penicillin G (mg/L)	Relative MIC fold change	2Identified mutations in PBPs
DA17021	FtsI	Hydroxylamine	12	2.4	P311S
DA17023	FtsI	Hydroxylamine	13	2.6	V545I
DA17025	FtsI	Hydroxylamine	8	1.6	V377I
DA20073	FtsI	ep-PCR	22	4.4	V545I, N579K, Syn N324 (AAC->AAT)
DA20075	FtsI	ep-PCR	24	4.8	V545I, P50S
DA20078	FtsI	ep-PCR	14	2.8	P311S, A49V, I159V
DA19904	DacC	ep-PCR	13	2.2	M369L, Syn V374 (GTC->GTT), W384C
DA19906	DacC	ep-PCR	12	2	S6Y, P199S, Syn A242 (GCT->GCA), Q365H, L368F
DA19908	DacC	ep-PCR	14	2.3	F21L, S120T, Syn L330 (CTG->CTA)
DA19909	DacC	ep-PCR	16	2.7	M45I, A104T, S147N, Syn S49 (AGC->AGT), Syn A141 (GCC->GCT)
DA19911	DacC	ep-PCR	13	2.2	T92M, Syn D294 (GAC->GAT), G325W,
DA19912	DacC	ep-PCR	21	3.5	Syn G116 (GGC->GGT), G146R, Syn L368 (TTA->TTG), M371I
DA20082	DacB	ep-PCR	10	1.7	Syn A34 (GCC->GCT), M39V, V66E, P78L, Syn T85 (ACG->ACA), Syn T110 (ACG->ACA), Syn L123 (CTG->TTG) G127C, W151Stop, T157M, V192M, Syn S194 (TCT->TCG), T278M, Syn L298 (CTG->TTG), I318T, N350I, T351P, G356D, Syn R402 (CGC->CGG), A403T, Q455L, Y474Stop
DA20084	DacB	ep-PCR	8	1.3	M1R, Q114L, K147Stop, R205Q, D312V, M371I

1 MIC values were determined under 2 mM arabinose induction.

2 Syn  =  synonymous mutations.

For the arabinose-dependent mutants isolated from the dacB_HM and dacC_HM libraries, the resistance was probably conferred by overexpression of the wild type PBP gene combined with other chromosomal mutations. Failure to isolate *dacB* or *dacC* variants conferring increased resistance against penicillin G was most likely due to the fact that more than one mutation is required to confer resistance and most PBP variants in the hydroxylamine generated libraries only contained one nucleotide change. To test this hypothesis, two mutagenesis libraries, designated dacB_epPCR and dacC_epPCR, were constructed by error prone PCR. The ribosome binding site and coding region of the *dacB* and *dacC* genes was amplified by error prone PCR at a 0.2% mutagenesis frequency. The amplified DNA was cloned into the pUCBAD-kan vector and the resulting library contained ∼2×10^6^ transformants. From the dacB_epPCR and dacC_epPCR libraries, six and two transformants, respectively, with ∼2- to 4-fold increases in resistance against penicillin G were selected (Table 2). Multiple amino-acid substitutions were identified in each of these variants, which support our previous hypothesis that in *dacB* and *dacC* more than one mutation are required to confer resistance (Table 2). Since both resistant *dacB* variants generated truncated proteins with many mutations, these two variants were excluded from further analysis.

To further explore the potential for the PBP *ftsI* to evolve increased resistance, two mutagenesis libraries, designated FtsI[P311S]_epPCR and FtsI[V545I]_epPCR, were constructed by performing error prone PCR on the two *ftsI* variants (FtsI[P311S] and FtsI[V545I]) that were isolated from the ftsI_HM library. The mutagenesis rate was estimated to be 0.2% and the resulting library contained ∼2×10^6^ variants. One resistant transformant with two additional substitutions was isolated from the library FtsI[P311S]_epPCR and the resistance level was only slightly increased compared to the parental variant with the single substitution P311S. Two resistant transformants, each of which contained one additional substitution, were isolated from the library FtsI[V545I]_epPCR and the resistance against penicillin G was increased ∼2-fold compared to the parental variant with the single substitution V545I.

Cross-resistance to four other β-lactam antibiotics was determined for six transformants selected from the *ftsI*-based libraries and six transformants selected from the *dacC*-based library (Table 3). In general, all tested tranformants showed increased resistance against the other four β-lactam antibiotics. The exception to this general trend was that the resistance level against cefotaxime for transformants selected from the *ftsI*-based libraries was only slightly increased or unchanged compared to the wild type strain.

**Table 3:** Relative MIC fold change of resistant transformants obtained from the FtsI-based libraries and the DacC-based library against five different β-lactam antibiotics.

Strain No.	Mutant PBPs	Amino acid substitutions	Antibiotics*				
			PG	AMP	CE	XM	CT
DA16010	DacC	Wild type	1.0	1.0	1.0	1.0	1.0
DA19904	DacC	M369L, W384C	2.2	2.0	2.3	2.3	2.5
DA19906	DacC	S6Y, P199S, Q365H, L368F	2.0	1.5	2.3	2.3	2.0
DA19908	DacC	F21L, S120T	2.3	1.5	2.0	2.3	2.0
DA19909	DacC	M45I, A104T, S147N	2.7	2.0	2.3	2.8	2.0
DA19911	DacC	T92M, G325W	2.2	2.0	3.0	2.8	2.0
DA19912	DacC	G146R, M371I	3.5	1.5	3.0	2.3	2.5
DA16020	FtsI	Wild type	1.0	1.0	1.0	1.0	1.0
DA17021	FtsI	P311S	2.4	1.5	4.0	3.7	1.3
DA17023	FtsI	V545I	2.6	1.5	1.3	2.3	0.7
DA17025	FtsI	V377I	1.6	1.0	1.7	1.7	1.3
DA20073	FtsI	V545I, N579K	4.4	3.5	5.3	3.7	1.7
DA20075	FtsI	V545I, P50S	4.8	4.0	4.7	6.0	1.3
DA20078	FtsI	P311S, A49V, I159V	2.8	2.0	3.0	4.0	1.3

*MIC values were determined under 2 mM arabinose induction.

Abbreviations for antibiotics: PG, penicillin G; AMP, ampicillin; CE, cephalothin; XM, cefuroxime; CT, cefotaxime.

### Effect of individual mutations on increased resistance

Since all the resistant variants selected from the libraries FtsI[P311S]_epPCR, FtsI[V545I]_epPCR, and DacC_epPCR contain multiple amino-acid substitutions, to examine the influence of each of these substitutions on the increased resistance, FtsI mutants N579K, P50S, A49V, and I159V and DacC mutants M369L, W384C, S6Y, P199S, Q365H, L368F, F21L, S120T, M45I, A104T, S147N, T92M, G325W, G146R and M371I were produced by site-directed mutagenesis as described in Materials and Methods. The resulting plasmid was then prepared from *E. coli* cells and transformed into the *Salmonella typhimurium* LT2 wild type strain. MIC against penicillin G was determined for each of these 19 strains (Table 4). The only reconstructed single mutant that showed increased MIC value against penicillin G was the DacC mutant W384C and the level of increased resistance for this single mutant was as high as for the DacC variant M369L/W384C. Therefore, the mutation M369L is likely to be a hitchhiker without contributing to the increased resistance against penicillin-G. All other single mutants exhibited a resistance level similar to the strain carrying the wild type PBP. This indicated that the increased penicillin-G resistance in all the variants isolated from the ep-PCR based mutagenesis libraries except the DacC variant M369L/W384 was due to a combinational effect of the amino acid substitutions identified in these variants and genetic interactions existed between the substitutions. Another possibility is that the single mutation variants (in contrast to the multiple amino acid substitutions mutants) are recessive to the wild type. It is noteworthy that only one mutation was identified from each of the three FtsI variants isolated from the ftsI_HM library and therefore these three mutations (P311S, V545I and V377I) alone could confer increased resistance.

**Table 4:** MICs of pencillin G for the reconstructed single mutants.

Strain No.	Mutated PBPs	Single mutation	MIC of penicillin-G* (mg/L)
DA16020	FtsI	Wild type	5
DA22042	FtsI	N579K	6
DA22043	FtsI	P50S	4
DA22044	FtsI	A49V	4
DA22045	FtsI	I159V	4
DA16010	DacC	Wild type	6
DA25863	DacC	M369L	4
DA25864	DacC	W384C	12
DA25865	DacC	S6Y	4
DA22046	DacC	P199S	6
DA25866	DacC	Q365H	4
DA25867	DacC	L368F	4
DA25868	DacC	F21L	4
DA22047	DacC	S120T	4
DA22048	DacC	M45I	6
DA22049	DacC	A104T	4
DA22050	DacC	S147N	6
DA22051	DacC	T92M	6
DA25869	DacC	G325W	4
DA22052	DacC	G146R	4
DA25870	DacC	M371I	4

*MIC values were determined under 2 mM arabinose induction.

### Identification of domain locations of the mutations

**Figure 1:**
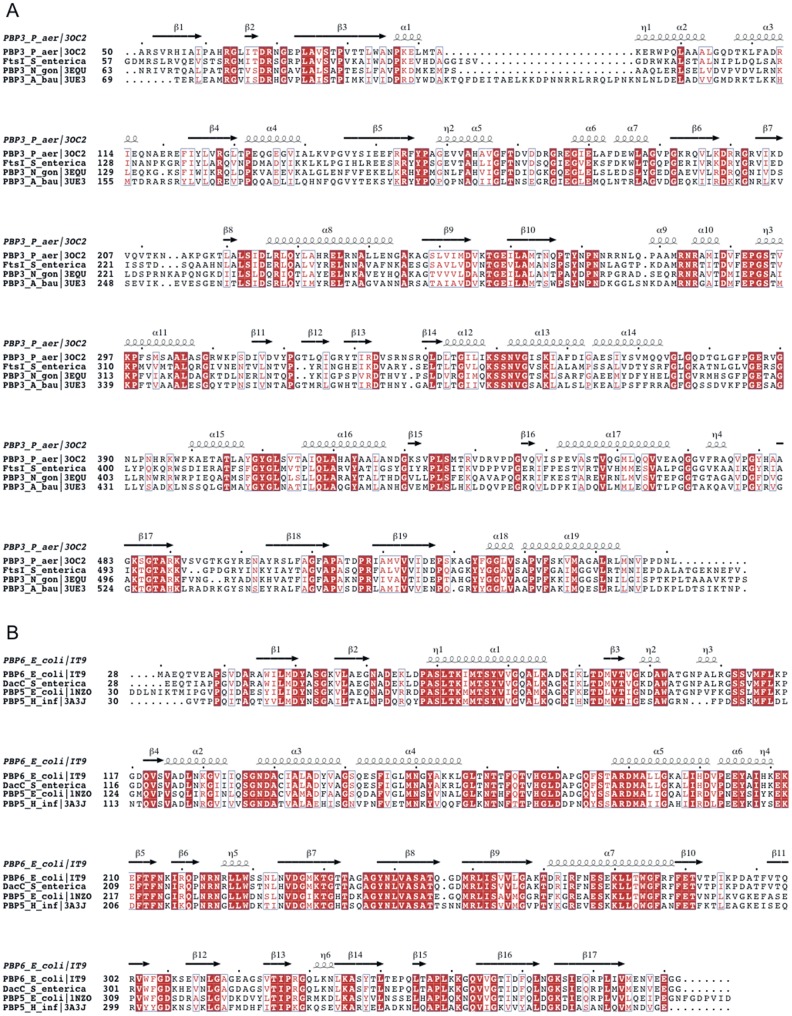
Sequence alignments of *S. enterica* FtsI (A) and DacC (B) with PBPs with known structure. Secondary structure is indicated above the alignment. Strictly conserved residues are shown in white on red background, residues with conserved physico-chemical properties are shown in red on white background. Mutation sites in this study are marked with asterisks below the alignment. The figure was prepared using ESPRIPT [34].

Penicillin-binding protein PBP3, which is encoded by the *ftsI* gene, is a high molecular mass class B PBP involved in the cell septation [23], [24]. In *Escherichia coli,* PBP3 is composed of a membrane anchor (amino acids 1–56), an N-terminal so-called non-penicillin-binding (n-PB) domain (amino acids 57–236), a C-terminal penicillin-binding (PB) transpeptidase domain (amino acids 237–577) and a cleavable signal peptide (amino acids 578–588) [24]. There are three conserved motifs of the penicilloyl serine transferases in the C-terminal PB module: STVK (amino acids 307–310), SSN (amino acids 359–361) and KTG (amino acids 494–496). The most similar proteins with known structures are PBP3 from *Pseudomonas aeruginosa* (PDB 3OC2 [25]) and *Acinetobacter baumanni* (PDB 3UE3 [26]) and PBP2 from *Neisseria gonorrhoeae* (PDB 3EQU, 3EQV, [27]) that all share more than 40% sequence identity with the *S. typhimurium* PBP3 (Figure 1A), indicating that these proteins have similar overall structure. Penicillin-binding protein PBP6 encoded by the *dacC* gene is one of the two primary DD-carboxypeptidases in *E. coli*, which are involved in moderating the degree of cross-linking of the cell wall [28]. As a member of low molecular mass PBPs, PBP6 is composed of an N-terminal signal peptide (amino acids 1–27), a PB domain (amino acids 28–284), a C-terminal domain of unknown function (amino acids 285–387) and a membrane anchor (amino acids 379–400) [29]. The three conserved sites of the PB module are SLTK (amino acids 66–69), SGN (amino acids 132–134) and KTG (amino acids 235–237). The crystal structure of *E. coli* PBP6 displays 95% sequence identity to *S. typhimurium* PBP6 and has been solved as an acyl-enzyme intermediate with the antibiotic ampicillin or a peptidoglycan substrate fragment [29]. Structures are also available for PBP5 from *E. coli* and *H. influenzae* [30], [31] with 65% sequence identity to *S. typhimurium* PBP6 (Figure 1B). The PBP3 and PBP6 in *Salmonella typhimurium* display 96% and 93% sequence identity to the respective *E. coli* counterparts and have the same length (Figure S1AB in File S1). Therefore we assume that the structures of *Salmonella typhimurium* PBP3 and PBP6 are very similar to the *E. coli* counterparts.

**Figure 2:**
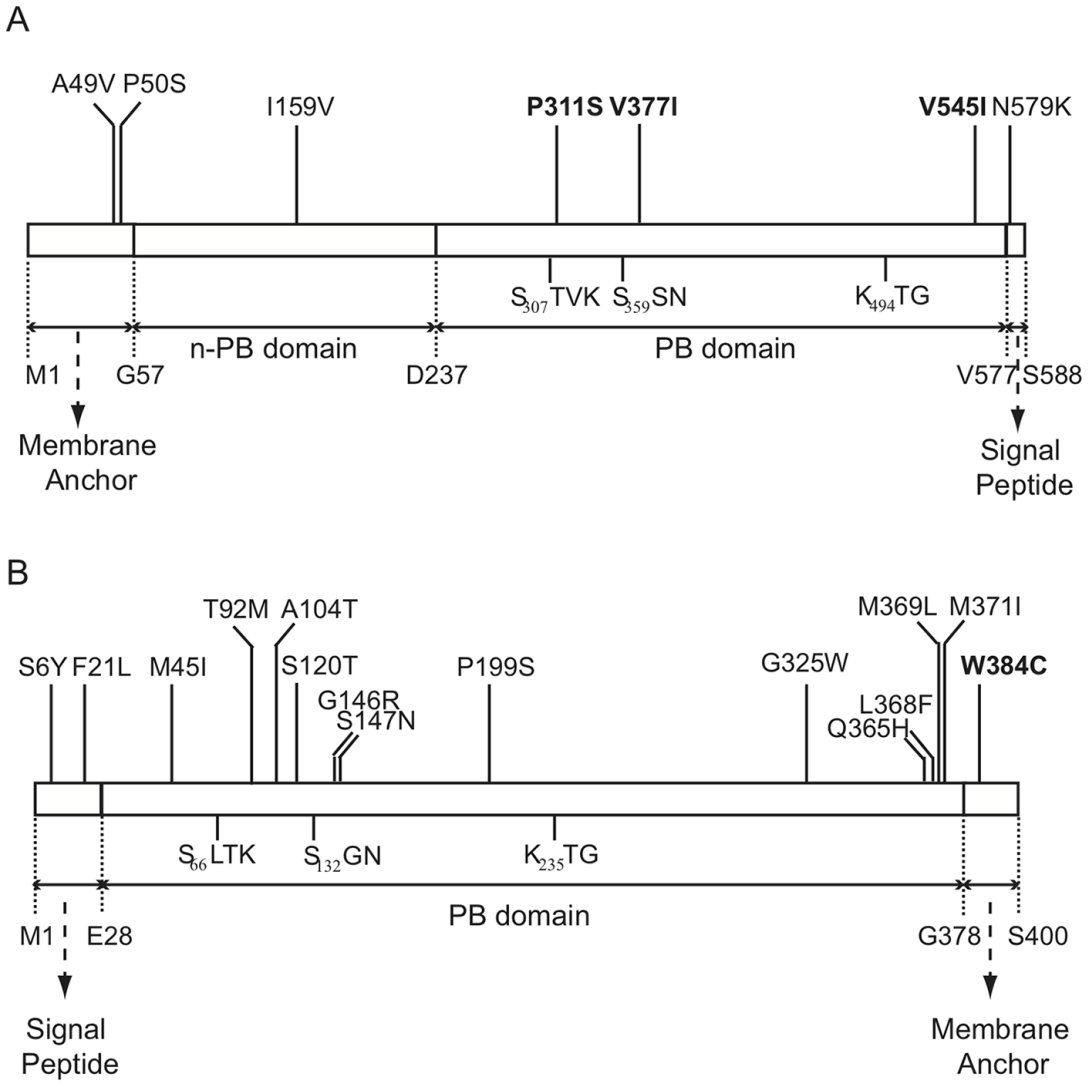
Domain locations of identified amino acid substitutions in the FtsI (PBP3) and DacC (PBP6) variants that conferred resistance against penicillin G. (A) Domain locations of mutations in PBP3. (B) Domain locations of mutations in PBP6. The three conserved sites of the PB module are shown. Mutations that, when present singly, confer resistance are indicated in bold.

The domain locations of the identified amino-acid substitutions in the resistant *ftsI* and *dacC* variants are shown in Figure 2. For the seven mutations identified from the *ftsI* variants, all the three first-step mutations, which were identified from the variants that were isolated from the hydroxylamine library, are located in the C-terminal PB domain that can catalyze acyl transfer reactions [32]. Among the other four second-step mutations identified from the variants that were isolated from the ep-PCR libraries, two are located in the membrane anchor-containing domain, one is located in the n-PB domain with an unknown function and one is located in signal peptide region. For the fifteen mutations identified from the *dacC* variants, twelve are located in the PB domain, two are located in the signal peptide region and one is located in the membrane anchor-containing domain.

### Structural prediction and analysis

**Figure 3:**
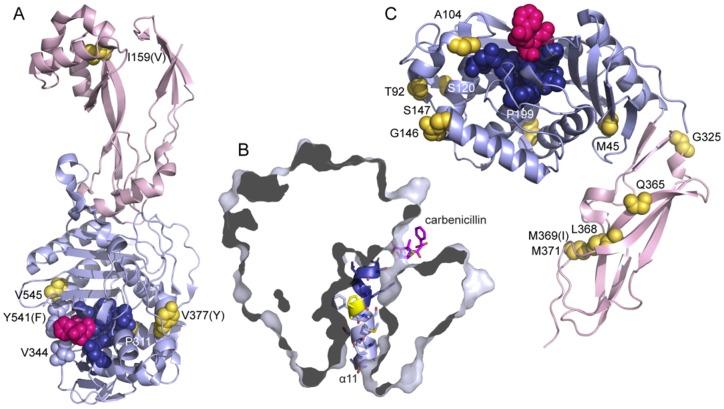
Location of mutation sites in known structures. **A.** Structure of PBP3 from *P. aeruginosa* (in complex with carbenicillin, pdb entry 3OCL (27)) The N-terminal n-PB domain is shown in pink and the C-terminal PB domain in pale blue. Residues are named and numbered according to the *S. typhimurium* FtsI sequence, for non-conserved residues the equivalent residue in *P. aeruginosa* is shown within parentheses. Carbenicillin (magenta), the catalytic motifs _307_STVK_310_, _359_SSN_361_ and _494_KS/TG_496_ (dark blue), residues equivalent to mutation sites in *S. typhimurium* DacC (gold, numbered) and residues V344 and Y541 are shown in spacefilling representation. **B.** P298 (yellow) creates a kink in helix α11 (cartoon and sidechains shown) that seems critical for interactions with the surrounding PB domain (surface representation). The STVK motif is shown in dark blue. **C.** Structure of PBP6 from *E. coli* (acyl-enzyme-intermediate with ampicillin, pdb entry [29]). The N-terminal PB domain is shown in pale blue and the C-terminal n-PB domain in pink. Residues are named and numbered according to the *S. typhimurium* DacC sequence, for non-conserved residues the equivalent residue in *E. coli* is shown within parentheses. Ampicillin (magenta), the catalytic motifs _66_SLTK_69_, _132_SGN_134_ and _235_KTG_237_ (dark blue) and residues equivalent to mutation sites in *S. typhimurium* DacC (gold, numbered) are shown in spacefilling representation.

A sequence alignment of *S. typhimurium* PBP3 with the most similar PBP3s and PBP2 of known structure (Figure 1A) shows that among the mutation sites, P311 and V545 are conserved, while I159 and V377 are variable. Based on this alignment and analysis of the available structures, we could locate mutation sites and predict the effects of the single-site mutations P311S and V545I in PBP3 (Figure 3A). P311 is a conserved residue that follows right after the catalytic STVK motif, where it induces a small kink in helix α11 (numbering according to *P. aeruginosa* PBP3, Figure 3B), suggesting that the P311S mutation will lead to straightening of the helix. On both sides of the kink, the helix packs against six surrounding helices, and the P311S mutation may affect these contacts. V545 is located in helix α18 that packs against the KTG motif and contributes to the penicillin-binding site in *P. aeruginosa* PBP3. V545 points away from the active site and contacts side chains from β19 and α8. In this region of the protein, the aromatic Y541 (F531 in *P. aeruginosa* PBP3, Figure 3A) changes its conformation upon substrate binding and closes the penicillin-binding site through an interaction with V333. The effect of V545I may be similar to D345a and P551S mutants in PBP2 from *N. gonorrhoeae* [27] and V547I in FtsI from *H. influenza* [33]. These mutations all seem to make changes that could affect this “closing” interaction. The double-mutants V545I/P50S and V545I/N579K display a higher level of resistance, but there is no structural information for the additional mutation sites.

Mutations in DacC occur in combinations of at least two different mutations. Despite the difficulty of predicting their combined effects, an analysis based on mainly the closely related *E. coli* PBP6 structure [29] gives some interesting hints.

In the PB domain, there are mutation sites in two structured turns, G146 and S147 between α3 and α4 and P199 between α5 and α6. Mutations of these residues as well as mutation of M45 in β1 (involved in hydrophobic packing) may affect the tertiary domain structure and thereby the position of catalytic motifs with respect to each other. A104T is located in η3 that is followed by a loop that hydrogen bonds to the SGN motif and contributes to the substrate-binding pocket. T92M and S120T are surface exposed and the role is less clear.

Notably, four of the DacC mutants have mutations in the C-terminal domain that forms a beta sandwich with unknown function and one of them is exclusively mutated in this domain and the membrane anchor. Several of these mutation sites (L368, M369, M371) are in the hydrophobic core of the domain, and mutations will likely have a de-stabilizing effect. T92M, S120T and Q365H will probably instead change the surface properties. Possibly, these mutations cause resistance through effects on interactions with other components; such a role of this domain has been suggested (13).

A novel resistance mechanism was recently demonstrated in PBP2a from *S. aureus.* Its so-called n-PB domain was shown to be an allosteric beta-lactam-binding domain where resistance mutations at a far distance from the active site act through disturbing an allosteric site (Otero et al., PNAS 2013). More commonly, mutations in PBPs can lead to resistance by two different mechanisms. The first one involves lowering the affinity of the drug to the active site, allowing the DD-transpeptidase activity to proceed without inhibition in presence of the drug [7], [18]. The second variant is to evolve beta-lactamase activity, *i.e.* to catalyze the deacylation reaction where water as a nucleophile attacks the scissile bond between the enzyme and the beta-lactam [5]. For this reason, the active sites of beta-lactamases are more exposed to solvent and contain a catalytic residue that activates a water molecule for the nucleophilic attack. The proposed catalytic residues are located in loops at the active site or in the same helix as the reactive serine [17]. None of the mutations identified in PBP3 introduce a new catalytic residue in this region. However, as described in detail above, we predict that the mutations P311S and V545I induce a more open catalytic site, and we hypothesize that the effect of this is to increase the accessibility for water, and/or to lower the affinity to beta-lactam antibiotics.

## Supporting Information

File S1 Figure S1AB, Amino acid sequence alignment of the PBP3 and PBP6 in *E. coli* and *Salmonella typhimurium*. (A) Alignment of the PBP3. (B) Alignment of the PBP6. Table S1, Oligonucleotide primers.
(DOCX)
