# Supplementary material for: Resistance to β-Lactam Antibiotics Conferred by Point Mutations in Penicillin-Binding Proteins PBP3, PBP4 and PBP6 in Salmonella enterica
Source: PLoS One. 2014 May 8;9(5):e97202. doi: 10.1371/journal.pone.0097202 (PMC4014608; doi:10.1371/journal.pone.0097202)
Supplement: File S1 — Figure S1AB, Amino acid sequence alignment of the PBP3 and PBP6 in E. coli and Salmonella typhimurium. (A) Alignment of the PBP3. (B) Alignment of the PBP6. Table S1, Oligonucleotide primers. (DOCX) [file pone.0097202.s001.docx]

**Resistance to ß-lactam antibiotics conferred by point mutations in penicillin- binding proteins PBP3, PBP4 and PBP6 in *Salmonella enterica***

Song Sun, Maria Selmer and Dan I. Andersson

**Supplementary information**

**TABLE S1.** Oligonucleotide primers.

| **Replacing the replication origin of pBAD30 with the pUC19 replication origin** | |
| --- | --- |
| pBAD30_up_F | CGATGCATAATGTGCCTGTC |
| pBAD30_dwon_R | AGCATATGTACAGGGCGCGTAAATCAA |
| pUC_up_F | TACATATGCTCATGACCAAAATCCCTTAAC |
| pUC_dwon_R | TTATGCATAGAACATGTGAGCAAAAGGCCA |
| **Replacing the TEM-1 ß-lactamase gene with the aminoglycoside 3’phosphotransferase gene** | |
| pBAD30_AmpR_downstream_F | CGACCTCGAGCTGTCAGACCAAGTTTACTCATATATA |
| pBAD30_AmpR_upstream_R | TTTTGCGGCCGCAAACAAAAGAGTTTGTAGAAACG |
| pKD4_Kan_clone_F | TCTTGCGGCCGCAAGATCCCCTCACGCTG |
| pKD4_Kan_clone_R | GGGTCTCGAGGAGCGCTTTTGAAGCTG |
| **Cloning PBPs in *Salmonella typhimurium* LT2** | |
| ampH_clone_F | TAGCGAATTCCTGCTTTTTACTGCCATGCT |
| ampH_clone_R | GCTCTCTAGATCAGGATGCAGGAATAGCAA |
| dacA_clone_F | TAGCGAATTCGTCGTAGTTCAGACCATGAA |
| dacA_clone_R | GCTCTCTAGATTATCCAAACCAGTGATGGA |
| dacB_clone_F | TAGCGAATTCTGTTAGCGCGAGATTATGC |
| dacB_clone_R | GCTCTCTAGATCAGTTATTCTGATAAATATCCTT |
| dacC_clone_F | TAGCGAATTCTACTCGGGTGGTACTATGAC |
| dacC_clone_R | GCTCTCTAGATTACGAGAACCAACTGCCAA |
| dacD_clone_F | TAGCGAATTCGACCATCCTGAGGACATGCT |
| dacD_clone_R | GCTCTCTAGATTACGCTTTATGCTGAAAATAGT |
| mrcA_clone_F | TAGCGAATTCTTATAAACTGCCCAAATGACAC |
| mrcA_clone_R | GCTCTCTAGATCAGAACAGTTCGTGTGTTTC |
| mrcB_clone_F | TAGCGAATTCTTGCGGAGAAAAAGCATG |
| mrcB_clone_R | GCTCTCTAGATTAATTGCCGCCGAACA |
| mrdA_clone_F | TAGCGAATTCAAGATTAAGCAGCGGATGAA |
| mrdA_clone_R | GCTCTCTAGATTATTGGTCCTCCGCC |
| pbpC_clone_F | TAGCGAATTCATTGTGACGCCTTAAATGAAC |
| pbpC_clone_R | GCTCTCTAGATTAACGTATTAATTCAAAATTAAC |
| pbpG_clone_F | TAGCGAATTCATCAGAAATCTCATCATGCTGA |
| pbpG_clone_R | GCTCTCTAGATTAATCGTTCTGTGCGGTT |
| STM1836_clone_F | TAGCGAATTCTCGGCTGGAGATCAGGTG |
| STM1836_clone_R | GCTCTCTAGATTACGAACCCGGAACCG |
| STM1910_clone_F | TAGCGAATTCGTTCAGGTACAGGTAATGACTTTTA |
| STM1910_clone_R | GCTCTCTAGACTATGGCGCCTGATCCG |
| STM2478_clone_F | TAGCGAATTCACTGAGGGAATCATCATGAAAT |
| STM2478_clone_R | GCTCTCTAGATTATTTTAAGGAACCATATATCTGGT |
| yfeL_clone_F | TAGCGAATTCTAAGAAAAAATCATAATGCCAG |
| yfel_clone_R | GCTCTCTAGATTATTGTTGTATTTGTTCCTGG |
| ftsI_clone_F | GGTACCCGGGGGATAAACGCGACGCAT |
| ftsI_clone_R | CGACTCTAGATTACGATCTGCCACCTGTTC |
| **Site-directed mutagenesis*** |  |
| ftsI_N579K_F | ATTCGTAATTAAACAAGGCGAGGGA |
| ftsI_N579K_R | TCATTTTTTTCGCCCGTTGC |
| ftsI_P50S_F | AGATTATCGCCTCGGATATGCTG |
| ftsI_P50S_R | GTAGCCACGCCGCGCGT |
| ftsI_A49V_F | TACAGATTATCGTCCCGGATATGCT |
| ftsI_A49V_R | GCCACGCCGCGCGTC |
| ftsI_I159V_F | AAACTGCCGGGGGTTCATTTGCGT |
| ftsI_I159V_R | GAGTTTTTTGATGTAGTCCGCCATG |
| dacC_P199S_F | ATTCACGATGTGTCTGACGAATACG |
| dacC_P199S_R | CAAGGCCTTGCCGAGCAACGC |
| dacC_S120T_F | GGCGATCAGGTGACGGTTGCGGAT |
| dacC_S120T_R | TGGCTTCAGAAACATCACCGAC |
| dacC_M45I_F | CCTGGATCTTAATTGATTACGCCAG |
| dacC_M45I_R | CTCGCGCATCAACGCC |
| dacC_A104T_F | ACGGGGAACCCGACGCTACGTGGG |
| dacC_A104T_R | CGCCCAGGCATCTTTGC |
| dacC_S147N_F | ACGTCGCCGGAAACCAGGAGTCCTT |
| dacC_S147N_R | AGTCGGCAAGCGCGATAC |
| dacC_T92M_F | CCGATATGGTGATGGTGGGCAAAGA |
| dacC_T92M_R | TCAGCTTGATTTTCCCTGCCTT |
| dacC_G146R_F | GACTACGTCGCCCGAAGCCAGGAGT |
| dacC_G146R_R | GGCAAGCGCGATACAGGC |
| dacC_M369L_F | CAACGCCCGTTATTGGTGATGGAAA |
| dacC_M369_R | CTCAATGGATTTTCCGTTAAGCTG |
| dacC_W384C_F | CAGCCGGATGTGTGATTTTGTGCTG |
| dacC_W384C_R | AAAAATCCCCCCTCCTCGAC |
| dacC_S6Y_F | CCTCGCGGCAGGTTCG |
| dacC_S6Y_R | CTACGAAGGGAATAAGCGTATTGCG |
| dacC_Q365H_F | ATCCATTGAGCATCGCCCGTTAATG |
| dacC_Q365H_R | TTTCCGTTAAGCTGGAAATCGATG |
| dacC_L368F_F | CAACGCCCGTTCATGGTGATGGA |
| dacC_L368F_R | CTCAATGGATTTTCCGTTAAGCTG |
| dacC_F21L_F | TAAAGGCAGAGGAACAAACGATCG |
| dacC_F21L_R | CAGGCGAAGCGAGAAGGAATAAGAG |
| dacC_G325W_F | ACGATTCCGCGTTGGCAGCTTAAAA |
| dacC_G325W_R | CACAGACCCCGCATCGC |
| dacC_M371I_F | GTTAATGGTGATTGAAAACGTCGAG |
| dacC_M371I_R | GGGCGTTGCTCAATGGATT |

*All primers used for site-directed mutagenesis was 5’ phosphorylated.

**FIGURE S1AB.** Amino acid sequence alignment of the PBP3 and PBP6 in *E. coli* and *Salmonella typhimurium*. (A) Alignment of the PBP3. (B) Alignment of the PBP6.
